# COPD awareness in the urban slums and rural areas around Pune city in India

**DOI:** 10.1038/s41533-021-00220-4

**Published:** 2021-02-11

**Authors:** Deesha Deepak Ghorpade, Anchala Raghupathy, Jyoti Deepak Londhe, Sapna Jitendra Madas, Nisha Vijay Kale, Narula Arvinder Pal Singh, Reshma Sudhir Patil, Monica Sumit Barne, Prakash Prabhakar Rao Doke, Sundeep Santosh Salvi

**Affiliations:** 1 Chest Research Foundation, Pune, India; 2 Savitribai Phule Pune University, Pune, India; 3 Bharti Vidyapeeth Deemed University Medical College, Pune, India

**Keywords:** Chronic obstructive pulmonary disease, Diseases

## Abstract

COPD is the second leading cause of death and disability adjusted life years (DALYs) in India, yet, it remains poorly recognized. We aimed to study the level of awareness of COPD in urban slums of Pune city in India and its neighboring rural areas. All male and female subjects above the age of 30 years residing in 13 randomly selected slums of Pune city (total population of 3000) and 7 randomly selected neighboring rural villages (total population of 3000) were invited to participate in this cross-sectional community survey. After obtaining written informed consent, 13 trained community health workers (CHWs) administered a questionnaire that captured their level of awareness of COPD. Of the 6000 subjects approached, 5420 residents (mean age ± SD = 48.0 ± 13.5 years; 38% males) consented and answered all questions. The number of people who had ever heard the word COPD was 49/5420 [0.9% (0.6–1.1%); 0.7% (0.5–1.3%) of the urban slum dwellers and 1.15% (0.5–1.3%) of rural residents]. Among those who had never heard the word COPD (*n* = 5371), when asked what was the name of the disease caused by long-term tobacco smoking, 38% said cancer, 16.7% said asthma, and 4.4% said TB. Among those who had heard the word COPD *(n* = 49), 6.1% said it was a disease of the heart, and 61% attributed COPD to smoke and dust pollution and 20% to tobacco smoking. The level of awareness of COPD in the Indian community is extremely low, highlighting the need to have nationwide mass awareness programs in India.

## Introduction

Chronic obstructive pulmonary disease (COPD) is the second leading cause of death in the world and affects an estimated 300 million people, while in India it is the second leading cause of death and affects an estimated 53 million people^1^. According to the Global Burden of Disease (GBD) data, COPD causes more deaths and disability adjusted life years (DALYs) than malaria/TB, HIV-AIDS, and diabetes all put together^1^. This very high and growing burden of COPD in India is due to a large population that is exposed to a multitude risk factors such as tobacco smoking^2^, indoor exposure to biomass smoke^3,4^, burning of mosquito coils^5–7^, other indoor air pollutants^8^, out-door air pollution, post TB-COPD, and poorly treated chronic severe asthma. The economic burden of COPD in India is estimated to be around 6.2 billion USD every year^9^, although this is likely to be an underestimate.

Despite the huge and growing burden of COPD in India, more than 95% of COPD patients in the community remain undiagnosed^10^. Under-diagnosis leads to under-treatment, which contributes to further increase in morbidity and mortality. Although diagnosis is necessary to reduce the deaths and sufferings associated with COPD, but perhaps more importantly, prevention of COPD is more important in a resource poor country like India. A need for a National COPD Prevention and Control Program has been expressed^11^. One of the key components of a National Prevention and Control Program is to create awareness about the disease among the communities, for which we first need to understand the level of awareness in the community. To the best of our knowledge there are no studies in India that have evaluated the level of awareness of COPD in the community. The main objective of our study was to understand, if people in India have ever heard the word COPD and whether they are aware of the risk factors associated with COPD.

## Results

**Fig. 1:**
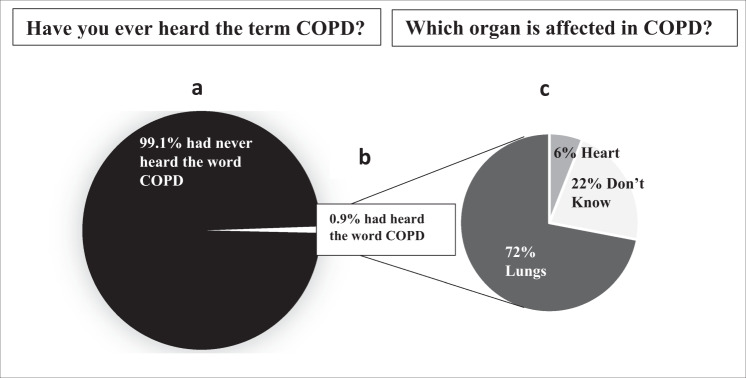
COPD awareness: “Have you ever heard the term COPD? Do you know what COPD means?” and “Which organ is affected in COPD?” Answers given by the 0.9% who had heard the word COPD. **a** Percentage of people who have never heard the term COPD. **b** Percentage of people who have heard the term COPD. **c** Responses to the question “Which organ is affected in COPD?” by the study participants who had heard the term COPD (0.9%).

Out of the 6000 people above the age of 30 years who were approached from 13 urban slums and 7 rural villages, 5420 consented to participate, and majority of the respondents were females (61%). The demographics have been explained in Table 1. To summarize the results of this study, we found that the level of awareness on COPD was very low both, in the urban slums and rural villages. Only 49 out of 5420 (0.9%) study participants said that they had ever heard the word COPD (0.7% from urban slum population and 1.15% from the rural village residents, and the difference between the two was not significant (*p* > 0.05) (Fig. 1). Among those who had heard the word COPD, 32 (65%) were males, 42 (86%) were never smokers, and 7 (14%) were ex-smokers or current smokers. In addition to those who had heard the word COPD, only 2 (4%) had a previous diagnosis of COPD. Among those who had heard the word COPD (*n* = 49), when asked “What is the organ that is affected in COPD?”, 71% (CI: 56–83%) answered lungs 6% (CI: 1–16%) answered heart, and 22% (CI: 11–36%) answered as did not know (Fig. 1). When asked “What are the causes of COPD?” 61% (CI: 46–74%) said air pollution/smoke and dust, 20.4% (CI: 10–34%) said tobacco smoking, 14.3% (CI: 5–27%) said don’t know, 2% (CI: 0.05–10%) said asthma, and 2% (CI: 0.05–10%) said tuberculosis. Participants who said they had never heard the term COPD when asked “What is the name of the disease caused by long-term tobacco smoking?”, 41% (CI: 39–42%) answered don’t know, 38% (CI: 36–39%) said cancer, 16% (CI: 15–17%) said asthma, and 4% (CI: 3–5%) said tuberculosis (Table 2).

**Table 1:** Demographic details describing the study population.

Demographic variables	Urban slums (*n* = 2993)	Rural villages (*n* = 2427)
Age (mean) ± SD (years)	48.4 ± 13.7	47.6 ± 13.3
Gender		
M:F	38%: 62%	40%: 60%
Education % (*n*)		
• Illiterate	27.3% (818)	20.8% (504)
• Primary school	15.8% (474)	17.1% (416)
• Secondary school	44.9 (1344)	51.3% (1244)
• Bachelor’s degree/Diploma	11.3% (337)	9.2% (223)
• Master’s degree	0.7% (20)	1.6% (40)
Tobacco smoking % (*n*)		
• Ex-smoker	5.8% (174)	3.6% (88)
• Current smoker	10.0% (302)	1.2% (29)
Biomass exposure % (*n*)	50.6% (1514)	76.7% (1862)
Work in a dusty occupation % (*n*)		
• Farming	4.6% (140)	48.6% (1180)
• Animal farming	10.7% (321)	23.8% (578)
Use of mosquito coils at home % (*n*)	25.7% (770)	36.1% (877)
Use of incense sticks % (*n*)	76.5% (2289)	75.5% (1832)

**Table 2:** Results of the responses for the four questions on COPD awareness that were asked to the study population.

Have you ever heard the term COPD? Do you know what COPD means?	Urban (*n* = 2993)		Rural (*n* = 2427)	
	Frequency	Percentage	Frequency	Percentage
Yes	21	0.70	28	1.15
No	2972	99.30	2399	98.85

if Yes, name of organ that is affected	Urban (*n* = 21)		Rural (*n* = 28)	
	Frequency	Percentage	Frequency	Percentage
Heart	0	0.0	3	10.7
Lungs	15	71.4	20	71.4
Don’t know	6	28.6	5	17.8

If yes, what are the causes of COPD?				
Causes of COPD	Urban (*n* = 21)		Rural (*n* = 28)	
	Frequency	Percentage	Frequency	Percentage
Asthma	1	4.7	0	0.0
Dust/smoke/pollution/smoke dust	14	66.7	16	57.1
Smoking/tobacco smoking	3	14.3	7	25.00
TB or lungs damage	1	4.7	0	0.0
Don’t know	2	9.5	5	17.8

If no, what is the name of the disease that is caused by long-term tobacco smoking?	Urban (*n* = 2972)		Rural (*n* = 2399)	
	Frequency	Percentage	Frequency	Percentage
Don’t know	1109	37.3	1100	45.8
Asthma	787	26.5	114	4.7
Cancer	882	29.7	1155	48.1
Tuberculosis	193	6.5	45	1.8

## Discussion

Among the 5420 residents from urban slums of Pune city and its neighboring rural villages who participated in this study, only 0.9% had ever heard the word COPD. There was no significant difference in the level of awareness for COPD between urban slum residents and those residing in rural villages. To the best of our knowledge, this is the first study in India that examined the level of awareness of COPD among residents of urban slum and rural villages. The fact that less than 1% of the community had ever heard the word COPD highlights the tremendous lack of awareness of COPD in the Indian community.

We conducted the study only among residents from urban slums and rural villages, primarily because COPD is associated with poor socioeconomic status and secondly, urban slums and rural villages are served by CHWs called Anganwadi and ASHA workers, respectively, who usually live within the communities and are well known to the people. It was therefore operationally easier to conduct house-to-house surveys in these populations than among people residing in the urban, non-slum population. Had we conducted the survey among the urban, non-slum population (areas not categorized as slums in the urban cities) in Pune city, we may likely have reported relatively higher proportions of residents who would have ever heard the word COPD, but this is still a speculation. Pune is the second largest district^12^ among India’s 732 districts and houses 58% of the population in the urban areas and 42% in the rural villages. Approximately 40% of the urban population resides in slums. Our study population (urban slums and rural villages) therefore represents over 65% of the district’s population. The low, almost negligible level of awareness about COPD in the study population in India is a matter of serious concern. The literacy rate in Pune district is 86% as compared to the national average of 74% and despite being a fairly literate district, the level of awareness was still very low.

There are at least eight such studies that have examined the level of awareness of COPD in the community, some from English speaking and some from non-English speaking countries. These studies have reported awareness rates of 49% in Turkey^13^, 21% in Japan^14^, 17% in Spain^15^, 8% in France^16^, 17% in Canada^17^, 4% in Brazil^18^, 10% in Germany^18^, and 1% in Korea^18^. India clearly stands out as a country with the lowest level of awareness of COPD in the community. Other diseases such as TB and HIV-AIDS are also English acronyms for tuberculosis and human immune deficiency virus—acquired immune deficiency syndrome respectively, but these are widely recognized across the Indian communities because of the tremendous awareness drives undertaken by the national and state governments in partnership with the WHO, non-government organizations, and the media. TB and AIDS are both household names in India and most people know the risk factors associated with these diseases although more than 99% may not even know the full form of these diseases. These awareness drives were a major contributor that led to a significant decrease in the burden of both TB and HIV-AIDS in India. Although India houses the world’s second largest English-speaking population (125 million), this constitutes only 10% of the country’s population. The 1.3 billion people in India speak 179 major languages and 544 dialects. It is possible that if the name “COPD” was given a local name in the local language, the level of awareness would have been higher. But having several hundred different names for COPD would have been completely confusing from a national health program perspective. It was therefore imperative to retain the standard uniform English name for COPD. It is now the responsibility of the policy makers and health educators in India to create the same level of awareness for COPD as they did earlier for TB and AIDS. The only way to tackle the low level of awareness of COPD is to bring about innovative ways of creating a long-standing impact in the communities. This could be achieved by proper structure and planning of feasible interventions, leading to creative outputs through social media channels, public relationship releases, and strategic communication in the targeted communities and at-risk populations. This can be followed by needs and impact assessment of the awareness campaigns. Strong impact-based awareness interventions need to be designed by considering various factors viz. lifestyle, belief systems, and attitudes that could affect the awareness outcomes.

In summary, the level of awareness of COPD in the Indian community is extremely low (<1%). There is an urgent need to create awareness drives using innovative means to start reducing the health burden of COPD which is currently the second leading cause of deaths and DALYs in India.

Our study in the urban slums and rural villages of Pune district has highlighted an important fact that <1% of people in the community have heard the word COPD. Furthermore, among the 99% who have never heard the term COPD, most people labeled tobacco-smoking-related diseases as cancer or asthma. This level of awareness about COPD is likely to be similar across other districts in India. COPD remains the second leading cause of death and DALYs in India, yet the awareness of the disease is so low that one of the first and fundamental steps in reducing the burden of COPD in India should be to start creating awareness through Public Health Programs. It is now time for all stake holders in India to stand up, take note, and act in this direction.

## Methods

### Study design

This study was a part of a larger study, which aimed at developing a COPD screening questionnaire to be used in the community by CHWs, by administering a set of questions, performing peak flow metry followed by pre and post bronchodilator spirometry. The C**O**PD **sc**reening questionn**a**i**r**e (OSCAR) study captured data on the awareness of COPD, demographic details, symptoms, risk factors, and co-morbid conditions associated with COPD. In this study, we report only the level of awareness of COPD among the 6000 residents whom we approached from urban slums and rural villages.

### Study population

Male and female subjects above the age of 30 years residing in the urban slums and neighboring villages and who were willing to take part in the study were the study population. Out of these six urban slums and six rural villages were randomly selected first from the list of slum areas and rural villages, but later we added seven more urban slums and one rural village which were randomly selected because we did not reach the requisite sample size.

### Study location

The study was conducted in the urban slums and rural villages of Pune district, which is the second largest district in India in terms of population (9.4 million). The Community Medicine Department of the Collaborator Institute have an existing social outreach program in 13 urban slums of Pune city (3.12 million) and 10 rural villages located in the outskirts of Pune city.

### Sample size

The sample size for this study was estimated based on the COPD prevalence of 6% with an absolute precision of 4.5% and a Type I error (*α*) of 0.05. The estimated sample size was 5167, which was increased to 6000 considering a drop-out rate of 15–20%, i.e. 3000 from the urban slums and 3000 from rural villages of Pune district. This sample size was similar to the sample size in the Spanish study which was similar to our study^15^.

### Research tool

A self-developed interviewee-administered questionnaire, which had four simple and straightforward questions on COPD awareness, was the research tool which were collected at one given point of time. The questionnaires were administered by the CHWs on a tablet (iBall Slide Nimble 4GF Tablet) using an electronic case reporting form (e-CRF) Version 4.1.8.

The questionnaire was first drafted in English and then translated into two local languages, viz. Marathi and Hindi, the most commonly spoken languages in this region and then further validated in a group of 10 patients with respiratory symptoms and 30 healthy subjects visiting the outpatient department as well as in the urban slum and rural village communities after obtaining a written informed consent. Minor modifications were suggested by external experts and the four final questions included (1) “Have you heard the word COPD?” If answered “yes”, they were asked, (2) “What are the causes of COPD?”, and (3) “Which organ is affected?”. Those who answered “No” were asked (4) “What is the name of the disease that is caused by long-term tobacco smoking?”

### Field staff and training

A total of 17 field staff were recruited for the data collection, which included 12 CHWs (6 Accredited Social Health Activist’s—(ASHA’s) for the rural villages and 6 Anganwadi workers for the urban slum dwellers) for administering the questionnaire, 4 field supervisors conducted quality checks on the field, and 1 Ph.D. Scholar (D.D.G.) managed the overall project. All the 12 CHWs resided in the study location. They were trained for 1 month to collect data on a tablet (iBall Slide Nimble 4GF Tablet) and to obtain informed consent from all the participants and in case of participants who were unable to read and write, consent was taken from an LAR (legally authorized representative), and administer the questionnaire to the study population. Mock interviews were conducted and the quality of data checked for errors. Only after the CHWs were confident to obtain informed consent and administer the questionnaire, were they sent on the field for data collection. The data were directly captured on to the tablet and transferred daily on to the laptop for further management.

### Quality checks on field

The field supervisors made surprise visits to randomly selected households where the CHWs had administered the questionnaire and examined the correctness of the survey interview. Data collected on the tablet were extracted at the back end in an excel format. The data manager then looked for presence of missing data. On receiving the list of missing data or unanswered data, if any, the CHWs were asked to revisit the houses or make telephonic calls, if the residents had a phone number and the missing data were collected. The complete and clean data were locked for further statistical analysis.

### Statistical analysis

Simple descriptive analyses, i.e. frequency, mean, median and standard deviation, interquartile range, minimum and maximum value were calculated using SPSS version 23.

### Ethics approval

The study was approved by the Institutional Ethics committee at the Chest Research Foundation, i.e. the Chest Research Foundation Institutional Ethics committee. All the documents viz. questionnaires, informed consent form, and subject information sheet were approved by the above-mentioned Institutional Ethics committee and we have complied with all relevant ethical regulations.

## Data Availability

The datasets generated during and/or analyzed during the current study are available from the corresponding author on reasonable request.
